# Changes in Aβ non-nociceptive primary sensory neurons in a rat model of osteoarthritis pain

**DOI:** 10.1186/1744-8069-6-37

**Published:** 2010-07-01

**Authors:** Qi Wu, James L Henry

**Affiliations:** 1 Psychiatry and Behavioral Neurosciences, McMaster University, 1200 Main St. West, HSC 4N35, Hamilton, Ontario, L8N 3Z5, Canada

## Abstract

**Background:**

Pain is a major debilitating factor in osteoarthritis (OA), yet few mechanism-based therapies are available. To address the need to understand underlying mechanisms the aim of the present study was to determine changes in sensory neurons in an animal model of OA pain.

**Results:**

The model displayed typical osteoarthritis pathology characterized by cartilage degeneration in the knee joint and also manifested knee pathophysiology (edema and increased vasculature permeability of the joint) and altered nociception of the affected limb (hind paw tenderness and knee articulation-evoked reduction in the tail flick latency). Neurons included in this report innervated regions throughout the entire hind limb. Aβ-fiber low threshold mechanoreceptors exhibited a slowing of the dynamics of action potential (AP) genesis, including wider AP duration and slower maximum rising rate, and muscle spindle neurons were the most affected subgroup. Only minor AP configuration changes were observed in either C- or Aδ-fiber nociceptors.

**Conclusion:**

Thus, at one month after induction of the OA model Aβ-fiber low threshold mechanoreceptors but not C- or Aδ-fiber nociceptors had undergone changes in electrophysiological properties. If these changes reflect a change in functional role of these neurons in primary afferent sensory processing, then Aβ-fiber non-nociceptive primary sensory neurons may be involved in the pathogenesis of OA pain. Further, it is important to point out that the patterns of the changes we observed are consistent with observations in models of peripheral neuropathy but not models of peripheral inflammation.

## Background

Osteoarthritis (OA) is the most common form of arthritis and it is a major health issue. Pain is the major complaint of patients with OA, and pain is reported to be more disabling than loss of joint function [1]. Various therapeutic approaches are available to treat OA pain, yet the effectiveness of existing drug therapies for OA pain is poor, with only moderate relief. A number of proposed mechanisms for OA pain have focussed on changes in knee joint nociceptors and their sensitivity. These mechanisms include activation of sensitized nociceptors in the knee by local inflammation [2-4], bone marrow lesions or micro fractures and increased intra-osseous pressure [5]. However, clinical data on OA suggest widespread changes in the properties of primary and secondary sensory neurons that might include low threshold knee joint afferents as well as neurons innervating tissues outside the joint. For example, most OA patients experience pain from the arthritic joint as well as referred pain from areas remote from the arthritic joint [6]. Further, even months following total hip replacement joint pain still persists in approximately 12% of patients [7]. Moreover, most OA patients also experience loss of proprioception [8-11] and loss of vibrational sense [12], which are mediated by Aβ-fiber low threshold neurons. This evidence suggests a general change in sensory neurons beyond simply changes in nociceptive neurons.

Changes in the functional properties of primary afferent neurons may be able to initiate these changes but they are largely overlooked as possible origins of the pain of OA, even though such changes have been suggested in other models of chronic pain, including models of inflammatory pain [13-15] and neuropathic pain [15-18]. Inflammatory pain models are associated with changes only in small dorsal root ganglion (DRG) neurons, possibly C- and Aδ-fiber neurons [13,19], which are usually associated with pain, or nociception. On the other hand, in neuropathic models all neuronal populations appear to be changed, including large Aβ-fiber neurons [20-27].

Therefore, the aim of the current study was to look beyond knee joint nociceptors and to investigate changes in sensory neurons within dorsal root ganglia (DRG) that innervate not only the knee but also neighboring DRG neurons that innervate other areas of the leg in an animal model of OA pain. In particular, we determined the electrophysiological properties of C- and Aδ-fiber nociceptive neurons as well as those of Aβ-fiber low threshold mechanoreceptors (LTMs), comparing these properties in naïve control animals and in OA animals one month after model induction. We report here that significant changes in action potential (AP) configuration were observed only in Aβ-fiber LTMs at a time during model development when the knee joint histopathology, knee pathophysiology and nociceptive responses of the affected limb confirmed that this was an animal model of OA that also exhibited altered sensory function. Only minor changes were observed in the small diameter nociceptive neurons. If changes in physiological properties reflect changes in functional role of these neurons in sensory processing, the present findings do not demonstrate changes in C- or Aδ-fiber nociceptors and therefore possible participation in the pathogenesis of pain in this surgically-induced rat model of OA.

## Materials and methods

This study was carried out on female Sprague Dawley rats (180-225 g) obtained from Charles River Inc. (Saint Constant, QC, Canada). All experimental procedures were approved by the McMaster University Animal Review Ethics Board and conform to the Guide to the Care and Use of Laboratory Animals of the Canadian Council of Animal Care, Vols.1 and 2, and experiments adhered to the guidelines of the Committee for Research and Ethical Issues of IASP published in PAIN^®^, 16(1983) 109-110.

Following model induction, animals were housed for one month before the acute electrophysiological experiment. At one month animals were tested for changes in nociceptive scores, and some animals were selected for histological and further physiological studies. After the end of the acute electrophysiological experiment each animal was euthanized by an overdose of anesthetic.

### Induction of the model of OA

Details of the surgical procedure to establish the surgical knee derangement model of OA have been reported previously [28]. In brief, animals were anesthetized with a ketamine-based anesthetic (ketamine, 100 mg/ml; xylazine, 20 mg/ml; and acepromazine, 10 mg/ml). The right side medial meniscus was removed, and the right side anterior cruciate ligament was cut to induce the unilateral OA. After surgery, the animals were given 0.05 ml of the antibiotic Trimel (sulfamethoxazole plus trimethoprim; Novopharm, Toronto, ON, Canada) once per day for 3 consecutive days, and the analgesic buprenorphine hydrochloride (Temgesic, Schering-Plough, Kenilworth, NJ, USA) twice per day for 2 consecutive days. Animals were allowed to survive for one month, as previous work in our group has suggested that typical signs of OA are entrenched by one month after surgery [29].

### Knee joint histopathology

Knee joints from the model animals were processed for histopathological evaluations in order to confirm that the model successfully mimicked OA. The knee joints were harvested and decalcified in 5% formic acid. Histological processing and assessment of tissues were done by Bolder BioPATH Inc. (Boulder, CO, USA). Briefly, knees were trimmed into two approximately equal frontal halves, processed through graded alcohols, and embedded in paraffin. An initial section was cut, and two additional step sections were cut at 150 μm for a total of three sections, which were stained with toluidine blue and evaluated microscopically for cartilage damage, osteophyte formation and the degree of joint instability. Cartilage degeneration in the tibia and femur was scored none to severe using the following criteria described by Janusz et al.: 1 = minimal superficial zone only; 2 = mild extends into the upper middle zone; 3 = moderate well into the middle zone; 4 = marked into the deep zone but not to tidemark; 5 = severe full thickness degeneration to tidemark [30]. Cartilage degeneration scores were measured for the medial and lateral tibia and femur, and all values from all three slides were summed to provide a total joint cartilage degeneration sum.

Osteophytes were scored 1, 2, 3, 4 or 5 for small (< 299 μm), moderate (300-399 μm), large (400-499 μm), very large (500-599 μm), or extremely large (> 600 μm) depending on the size using an ocular micrometer. Medial and lateral osteophyte scores were added to the total joint cartilage sum to derive a total joint score.

The degree of joint stability was subjectively determined based on fibroproliferative and chondrogenic changes in the synovium/collateral ligament as well as transected cruciate area: mild - some fibroplasia with minor proteoglycan deposition, small foci of chondrogenesis, marginal zone proliferation and/or small osteophytes; moderate - diffuse fibroplasia with proteoglycan deposition and larger foci of chondrogenesis, small to medium osteophytes; severe - diffuse severe thickening of synovium and ligaments with proteoglycan deposition and chondrogenesis, generally large osteophytes. The scoring system was as follows: mild = 1, moderate = 2, and severe = 3.

### Tissue edema and plasma extravasation of the knee joint

One group of rats was used to study pathophysiological changes associated with the joint capsule in this model, in particular to assay edema and vascular permeability. Briefly, knee joints were dissected and dried in an oven at 60°C for 24 h. Edema in the knee joint was determined as the weight difference of each knee joint after this dehydration procedure.

To evaluate vascular permeability the degree of plasma extravasation was determined. Evans blue dye (VWR, Mississauga, ON, Canada) was injected through the jugular vein. Twenty minutes following Evans blue dye injection, animals were perfused intracardially with 500 ml of physiological saline. Knee joints were harvested and dried as described above, and then were placed in vials each with 3 ml of formamide (Fisher Scientific, Ottawa, ON, Canada) overnight in an oven at 60°C. Twenty-four hours later, fluids in the vials were filtered and evaluated by the absorbance measured by color spectrophotometry (Biochrom Ltd., Cambridge, UK), compared to pure formamide at wavelength 620 nm. The optical density of Evans blue dye in the joint capsule tissue indicated plasma extravasation, and was calculated as follows: ipsilateral absorbance/ipsilateral weight.

### von Frey test to determine hind paw mechanical withdrawal threshold

To determine changes in nocifensive behaviors in OA animals von Frey test was conducted. Animals were placed in the testing chamber and allowed to acclimatize for 30 min prior to testing. von Frey filaments from Stoelting (Wood Dale, IL, USA) were applied to the soft tissue of the plantar surface of the hind paw to determine the withdrawal threshold [31]. The response pattern described by Chaplan was adopted to calculate 50% response threshold [32]. The maximum score possible was 15 grams, and the minimum was 0.25 grams.

### Effects of repeated flexion and extension of the OA knee on tail flick latency

Previously, we have established that noxious peripheral stimuli alter reaction time in the tail flick test [33-35]. Thus, we applied a similar approach to determine whether repeated flexion and extension of the deranged knee would be a noxious stimulus and therefore alter tail withdrawal latency.

Rats were gently wrapped in clean surgical drapes that covered the entire body to the base of the tail. They were acclimatized to the wrapping for 20-25 min, twice each day over a two-day period prior to surgery. Tail withdrawal latency was then determined on Model 33 tail flick Analgesia meter (IITC, Woodland Hills, CA, USA) at a point 10 cm from the tip that was blackened prior to the test. The intensity of light beam was set so that a baseline reaction time of 8-10 sec was achieved. Once stable baseline readings had been taken, the deranged knee was then articulated with a full extension-flexion mode through the normal plane of motion 20 times over a 30 sec period. Readings in the tail-flick test were then taken again 3 and 6 min after the articulation. The mean of the three baseline responses was taken as 100%. All subsequent responses were normalized as a percentage of the baseline value.

### Animal preparation for acute electrophysiological recordings

Full details of the animal preparation and intracellular recordings have been reported previously [28]. Recordings were made from the L4 DRG partly because it is one of the DRGs that contain knee joint afferents. In brief, the L_4 _dorsal root was cut close to the spinal cord to allow a 12-15 mm length for electrical stimulation. One pair of bipolar platinum stimulating electrodes (FHC, Bowdoinham, ME, USA) was placed beneath the L_4 _dorsal root.

The animals were ventilated to achieve an end-tidal CO_2 _concentration around 40 mmHg. Rectal temperature was maintained at approximately 37°C using an in-house servo-controlled infrared heating lamp. Immediately before the start of recording an initial 1 mg/kg dose of pancuronium was given to eliminate muscle tone. The effect of pancuronium was allowed to wear off hourly in order to confirm a surgical level of anesthesia by observing the pupil for dilation and testing for reflex withdrawal from a pinch to a forelimb. Throughout the experiments, supplemental pentobarbital was added hourly to maintain a surgical level of anesthesia.

Recordings were made intracellularly using sharp glass micropipettes with DC resistance around 40-70MΩ. The microelectrode was advanced with an EXFO IW-800 micromanipulator (Montreal, QC, Canada) until a resting membrane potential of at least -40 mV suddenly occurred and an AP could be evoked by stimulation of the dorsal root. Once this occurred the recording was allowed to stabilize over a five min period. Then, the stimulating electrode was used to deliver a single electrical pulse to evoke an AP for analysis. Recordings were made with a Multiclamp 700B amplifier (Molecular Devices, Union City, CA, USA) and digitized on-line via a Digidata 1322A interface (Molecular Devices) with *pClamp 9.2 *software (Molecular Devices). The first evoked AP in each neuron was used to determine any differences in configuration between control and OA animals. Measurements of the electrophysiological parameters have been demonstrated [28]. These included conduction velocity, resting membrane potential, AP duration at base, AP half width, AP amplitude, AP rise time, AP fall time, maximum rising rate, maximum falling rate, afterhyperpolarization amplitude, 50% afterhyperpolarization recovery time and 80% afterhyperpolarization recovery time. Analysis was done off-line using *pClamp 9.2 *software.

### Classification of DRG neurons

Response properties of neurons to natural stimuli of peripheral receptive fields were identified by various mechanical stimuli, and classified as previously described [36]. The criterion for the classification of C-, Aδ- and Aβ-fiber DRG neurons was mainly based on dorsal root conduction velocities: ≤ 0.8 m/s for C-fiber neurons, 1.5-6.5 m/s for Aδ-fiber neurons and ≥ 6.5 m/s for Aβ-fiber neurons [37].

The differentiation of nociceptive and non-nociceptive neurons was based specifically on their sensory properties identified during receptive field searching. Those responding to high intensity, potentially noxious, stimuli were classified as nociceptive neurons, whereas those responding to low intensity, innocuous stimuli were classified as non-nociceptive neurons.

Three major factors were considered in grouping Aβ-fiber LTMs: the threshold of activation, the depth of the receptive field and the pattern of adaptation. Non-nociceptive Aβ-fiber neurons were identified as low threshold mechanoreceptors using a soft brush, light pressure with a blunt probe and light manual tap. These neurons included various subtypes, such as guard hair, field hair, Pacinian, glabrous rapidly adapting, slowly adapting types I and II, and muscle spindle types I and II. Guard and field hair neurons were both rapidly adapting cutaneous hair units and are included together. Pacinian and glabrous neurons were both rapidly adapting non-hair neurons, and were named rapidly adapting neurons. Slowly adapting neurons adapted slowly to light tactile stimuli to the cutaneous receptive fields. Muscle spindle neurons were slowly adapting neurons with subcutaneous receptive fields

For C- and Aδ-fiber DRG neurons, only high threshold or unresponsive C- and Aδ-fiber neurons were recorded and included in the current electrophysiological study. High threshold neurons were those that were activated only by high intensity stimuli such as pinch and squeeze applied with a fine forceps, a coarse-toothed forceps or a sharp object such as a syringe needle. Unresponsive neurons were those not excited by any of the non-noxious or noxious mechanical stimuli listed above, and as defined by Lawson et al [36].

### Statistical analysis

Normality of electrophysiological data was done with the D'Agostino and Pearson omnibus test. Wherever appropriate, Student's *t*-test or the Mann-Whitney *U*-test was used for comparisons between OA and control animals in various neuronal subtypes and for various parameters. All statistical tests and graphing were done using Prism 4 software (GraphPad, La Jolla, CA, USA). *P*-values are indicated in the graphs and *P *< 0.05 was considered to indicate a statistically significant difference.

## Results

A total of 81 animals was used for different purposes in this study, 50 for electrophysiology (26 control and 24 OA animals), 17 for behavioral studies and knee joint pathology (7 control and 10 OA animals) and 14 for knee pathophysiological evaluations (7 control and 7 OA animals).

### Histopathological changes in the knee joint

**Figure 1:**
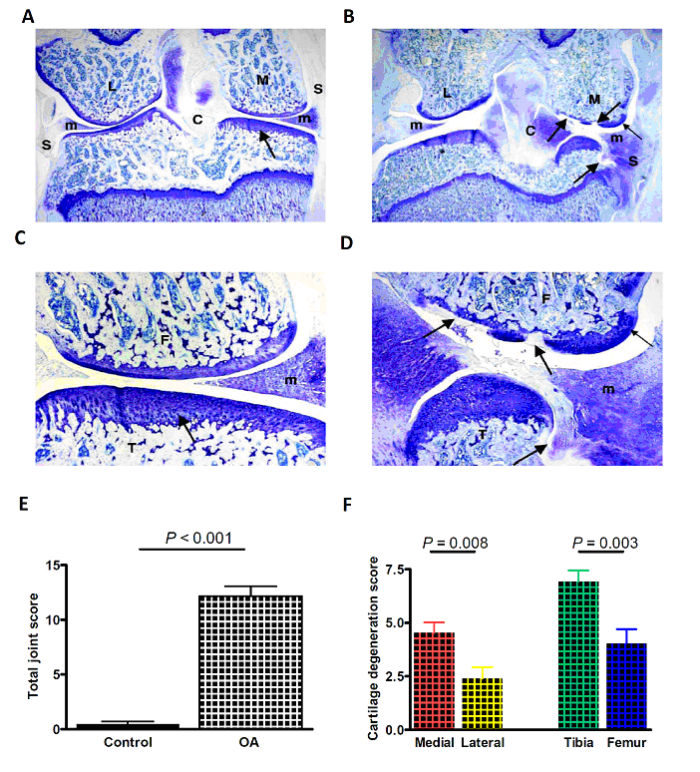
**Histology of the naïve control and OA knee joints**. **A, C**, knee from a naive animal has normal cartilage (arrows). Higher magnification of medial compartment from naive animal shows normal cartilage (arrows). **B, D**, knee from one month OA animal has significant cartilage degeneration on all articulating surfaces, with the greatest lesion severity on the medial femur (large arrows). There is severe atrophy of the medial tibia, as well as marked a reshaping of the medial tibial plateau and tibial epiphyseal bone. Additionally, there is a medium-sized osteophyte on the medial femur (small arrow). There is severe thickening/fibrous repair with proteoglycan on the medial side of the synovium and joint capsule. M = Medial; L = Lateral; S = Synovium; m = Meniscus; C = Cruciate ligaments. **E**, total joint score in OA knees (12.17 ± 0.9, *N *= 10) were significantly higher than in naïve control knees (0.47 ± 0.23; *N *= 7; *P *< 0.001; Mann-Whitney *U*-test). **F**, Medial cartilage degeneration was more severe than that of the lateral cartilage (4.53 ± 0.49, *N *= 10 vs. 2.4 ± 0.53, *N *= 10, respectively; *P *= 0.008; Student's *t*-test). Moreover, tibia cartilage degeneration was more severe than femur cartilage degeneration (4.53 ± 0.49, *N *= 10 vs. 2.4 ± 0.53, *N *= 10, respectively; *P *= 0.003; Student's *t*-test).

As determined by toluidine blue staining, knee joints in naïve control animals showed sporadic minimal cartilage degeneration on the inner part of the medial tibia, but without any osteophyte formation or any sign of joint instability (Fig. 1A, 1C). These minor changes are typical of common spontaneous medial tibial alterations in the cartilage area that is not protected by the meniscus. In animals with one month duration OA, lesions of the affected joint were observed, including cartilage degeneration ranging from superficial proteoglycan and chondrocyte loss (most joints) to focal marked to severe chondrocyte loss (less common, always medial; Fig. 1B). The medial and lateral cartilage degeneration sums were significantly increased, as well as the total joint score. Total joint scores of 0.5 ± 0.23 in naïve control knees (*N *= 7) were significantly lower than 12.2 ± 0.91 in OA knees (*N *= 10; *P *< 0.001; Fig. 1E). Moreover, the medial cartilage degeneration was more severe than lateral degeneration (4.5 ± 0.49, *N *= 10 vs. 2.4 ± 0.53, *N *= 10; *P *= 0.008; Fig. 1F). There was moderate to severe joint instability manifesting as varying amounts of damage to cruciate ligaments and medial menisci, as well as proliferative changes in both. The instability score was 2.3 ± 0.21 (*N *= 10). The medial joint capsule was thickened with proteoglycan deposition. The medial side of the joint typically exhibited the osteophyte formation. Some joints exhibited a reshaping of the medial tibial epiphyseal marginal zone and subchondral bone (Fig. 1D).

### Pathophysiological changes in the knee joint

**Figure 2:**
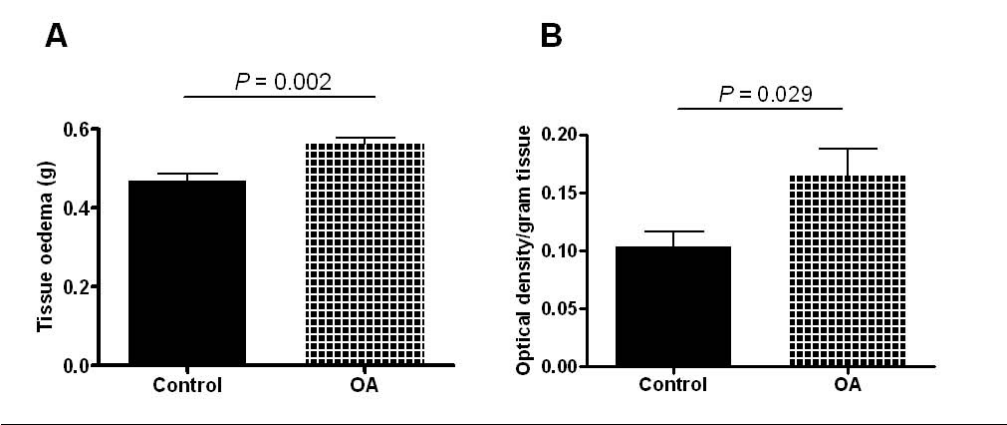
**Tissue edema and plasma extravasation of the knee joint in naïve control rats and OA rats**. **A**, significantly increased edema was found in OA knee joints (0.56 ± 0.02 g, *N *= 7 in OA vs. 0.47 ± 0.01 g, *N *= 7 in control; *P *= 0.002; Student's *t*-test). **B**, optical densities of Evans blue in knee joint soft tissue were significantly higher than that of the naïve control knee (0.17 ± 0.02 g, *N *= 7 in OA vs. 0.1 ± 0.01 g, *N *= 7 in control; *P *= 0.029; Student's *t*-test).

As determined by the weight difference after the dehydration protocol, OA rats exhibited significantly more liquid in the ipsilateral knee joint than control rats. The weight difference by dehydration per knee was 0.5 ± 0.02 g in naïve control rats (*N *= 7), and the amount was significantly increased to 0.6 ± 0.02 g in the ipsilateral knees in OA rats (*N *= 7; *P *= 0.002; Fig. 2A).

Extravasation of Evans blue dye, usually taken as a measurement of vascular permeability, was greater in OA rats. The optical density of Evans blue dye in knees from control rats was 0.1 ± 0.01 (*N *= 7), and was 0.2 ± 0.02 in OA rats (*N *= 7; *P *= 0.029; Fig. 2B), suggesting a loss of vascular integrity in the knees from OA animals.

### Nocifensive behaviors of the OA model

**Figure 3:**
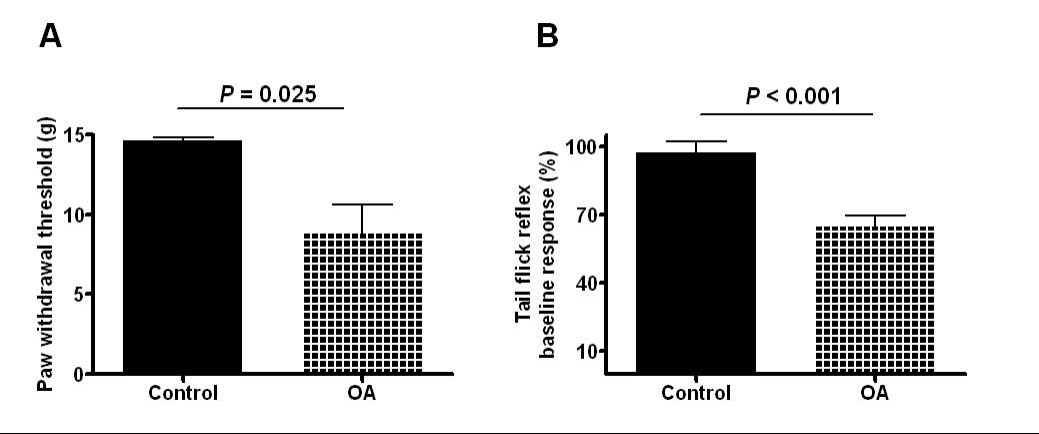
**Pronociceptive effects in sensory tests in OA models run one month following model induction**. **A**. OA rats displayed a significantly decreased threshold to the von Frey hair stimulation to the affected hind paw (8.78 ± 1.85 g, *N *= 9 in OA vs. 14.63 ± 0.23 g, *N *= 6 in control, *P *= 0.025; Student's *t*-test). **B**. Repetitive flexion and extension of the knee had no significant effect on the latency of the tail flick reflex in control rats (97.1 ± 5.2% of the baseline latency, *N *= 6). However, the same manipulation significantly decreased the latency of the tail flick reflex in OA rats (64.7 ± 4.9% of the baseline readings, *N *= 9; *P *< 0.001; Student's *t*-test).

Paw withdrawal thresholds were measured only at one month after surgical induction of the model. The threshold to von Frey hair stimulation in control animals was 14.6 ± 0.23 g (*N *= 6). The OA group showed significantly lower withdrawal thresholds, or higher sensitivity; the threshold was 8.8 ± 1.85 g (*N *= 9; *P *= 0.025; Fig. 3A).

The latency to withdrawal of the tail in the tail-flick test was also determined in these animals at one month after model induction. Repeated flexion and extension of the knee had no effect on the latency of the tail flick reflex in control rats (97.1 ± 5.18% of the baseline value). However, in OA rats the same manipulation significantly decreased the latency of the tail flick reflex to 64.7 ± 4.91% of the baseline reading (*P *< 0.001; Fig. 3B).

### AP configurations in C- and Aδ-fiber neurons

Acute electrophysiological experiments were run at one month after model induction.

The C-fiber pool was comprised of 24 neurons (5 neurons with an identifiable receptive field) from 14 OA rats and 32 neurons (19 neurons with an identifiable receptive field) from 21 control rats. The Aδ-neuron pool was comprised of 15 neurons (5 neurons with an identifiable receptive field) from 10 OA rats and 18 neurons (9 neurons with an identifiable receptive field) from 15 control rats.

**Figure 4:**
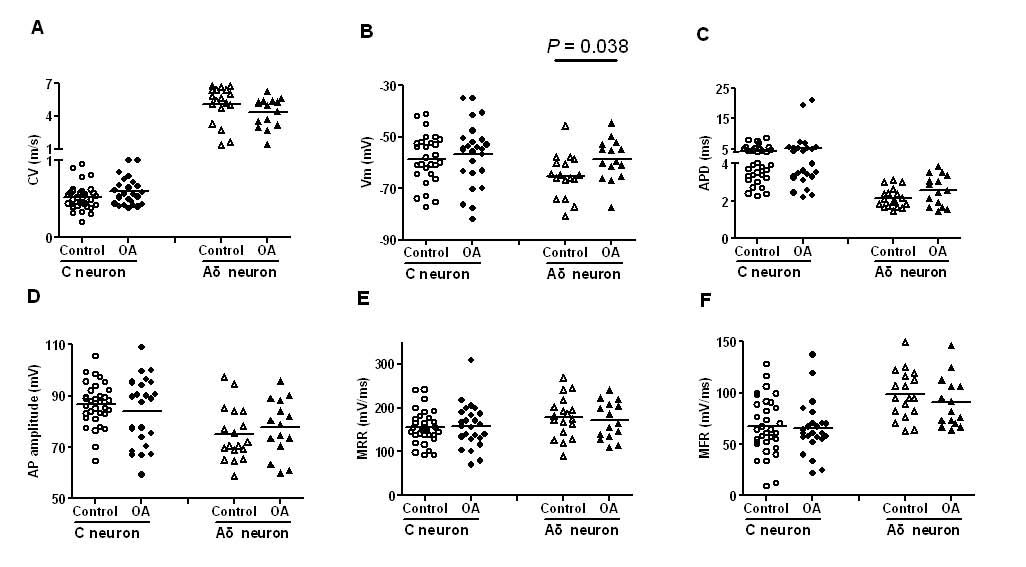
**C- and Aδ-fiber nociceptive DRG neurons**. Scatter plots indicating AP properties of individual neurons in control animals, and in OA animals at one month following model induction. "C neuron" stands for C-fiber DRG neurons which include C-fiber high threshold mechanoreceptors and C-fiber non-responsive neurons. Similarly, "Aδ neuron" represents Aδ-fiber DRG neurons which include Aδ-fiber high threshold mechanoreceptors and Aδ-fiber non-responsive neurons. The parameters that bear most of the documented changes are presented, including **A **AP duration at base, **B **AP rise time, **C **AP fall time, **D **AP amplitude, **E/F **maximum rising and falling rates. The only difference between OA and control rats was a more depolarized resting membrane potential in Aδ-fiber DRG neurons in model animals (-58.72 ± 2.11 mV; *N *= 15 in OA vs. -65.11 ± 2.04, *N *= 17 mV in control; *P *= 0.038; Student's *t-*test).

No difference between control and OA model rats was found in the conduction velocity in either C- or Aδ-fibers: 0.5 ± 0.03 m/s in control C-fiber neurons (*N *= 33) vs. 0.6 ± 0.04 m/s in OA C-fibers (*N *= 25; *P *= 0.099), and 5.1 ± 0.04 m/s in control Aδ -fibers (*N *= 18) vs. 4.4 ± 0.35 m/s in the OA Aδ-fiber neurons (*N *= 15; *P *= 0.219). C- and Aδ-fiber neurons appeared to conduct in two widely separated ranges (Fig. 4A).

The resting membrane potential in C-fiber neurons was similar in control rats (-58.7 ± 1.76 mV; *N *= 29) and in OA model rats (-56.7 ± 2.46 mV; *N *= 25; *P *= 0.497). However, resting membrane potential in Aδ-fibers in control rats (-65.1 ± 2.04 mV, *N *= 17) was less depolarized than in OA model rats (-58.7 ± 2.11 mV; *N *= 15; *P *= 0.038; Fig. 4B).

AP amplitude was similar in both control and OA rats (86.6 ± 1.49 mV, *N *= 33 in control C-fiber neurons vs. 84.1 ± 2.64 mV, *N *= 25 in OA C-fiber neurons; *P *= 0.387; 74.9 ± 2.48 mV, *N *= 18 in control Aδ-fiber neurons vs. 77.7 ± 2.85 mV, *N *= 15 in OA Aδ-fiber neurons; *P *= 0.474; Fig. 4D).

**Figure 5:**
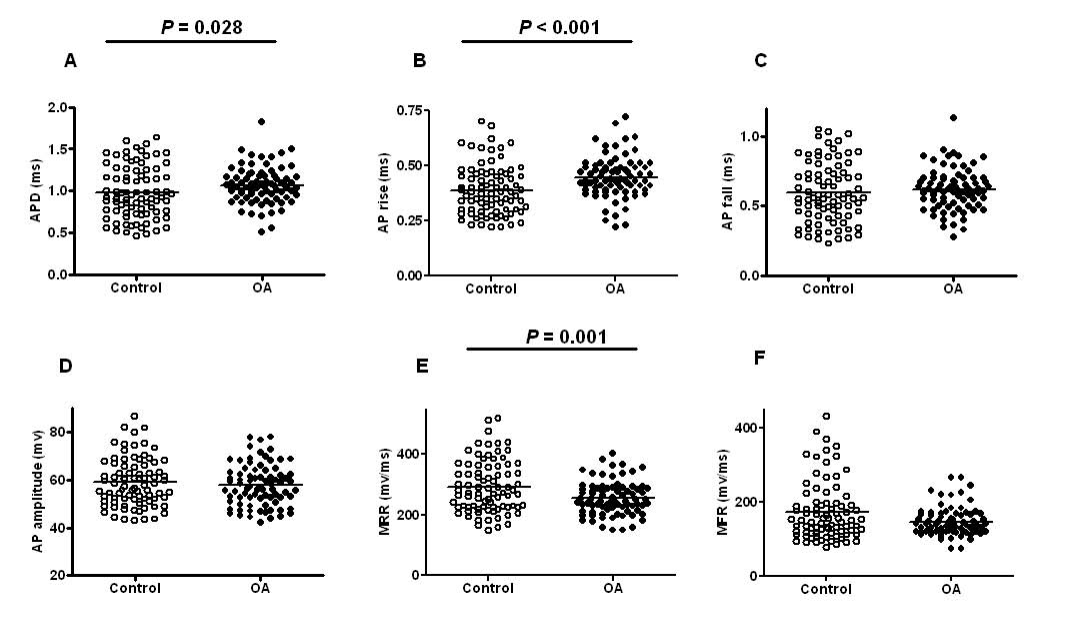
**Aβ-fiber low threshold mechanoreceptors**. Scatter plots of AP properties of individual neurons in control animals, and in OA animals at one month after model induction. The parameters that bear most of the documented changes are presented, including **A **AP duration at base, **B **AP rise time, **C **AP fall time, **D **AP amplitude, **E/F **maximum rising and falling rates. In each case the median (horizontal line) is superimposed. The D'Agostino and Pearson omnibus normality test was run in all data groups in order to assign the data to parametric or non-parametric *t *tests. APD (AP duration at base) was significantly wider in the OA group (1.07 ± 0.02 ms, *N *= 79 in OA vs. 0.98 ± 0.03 ms, *N *= 83 in control; *P *= 0.028; Mann-Whitney *U*-test). AP rise time was significantly longer in the OA group (0.45 ± 0.01 ms, *N *= 79 in OA vs. 0.39 ± 0.01 ms, *N *= 83 in control; *P *< 0.001; Student's *t*-test). MRR (Maximum rising rate) was significantly slower in the OA group (254.3 ± 6.22 mV/ms, *N *= 79 in OA vs. 291.9 ± 9.41 mV/ms, *N *= 83 in control; *P *= 0.001; Student's *t*-test).

The AP duration at base in C-fiber neurons in control rats (4.3 ± 0.28 ms; *N *= 33) was similar to that in OA model rats (5.4 ± 0.93 ms; *N *= 25; *P *= 0.789; Fig. 5C). It is also the case in Aδ-fiber neurons (2.2 ± 0.12 ms; *N *= 18 in control vs. 2.6 ± 0.21 ms; *N *= 15 in OA; *P *= 0.069; Fig. 4C). For the duration at half amplitude in C-fiber neurons, no difference was identified between OA (2.3 ± 0.33 ms; *N *= 24) and control rats (2.1 ± 0.14 ms; *N *= 33; *P *= 0.878), and no difference was found in Aδ-fiber neurons in control vs. OA rats (2.2 ± 0.12 ms; *N *= 18 and 2.6 ± 0.21 ms; *N *= 15, respectively; *P *= 0.156).

AP rise time reflects the duration of the depolarization phase of the AP. No significant difference between OA and control animals was found in the AP rise time in either C-fiber neurons or Aδ-fiber neurons. AP rise time in C-fiber neurons was 1.7 ± 0.14 ms in control (*N *= 33) vs. 2.1 ± 0.41 ms in OA (*N *= 25; *P *= 0.47); AP rise time in Aδ-fiber neurons was 0.9 ± 0.05 ms in control (*N *= 18) vs. 1.1 ± 0.09 ms in OA (*N *= 15; *P *= 0.095). Maximum rising rate was derived from the differentiated conversion of the AP curve, which reflects the rate of depolarization over time. The maximum rising rate in C-fiber neurons was similar in control and OA rats (154.5 ± 6.51 mV/ms in control, *N *= 33 vs. 158.1 ± 9.95 mV/ms in OA, *N *= 25; *P *= 0.796). Similar is maximum rising rate in Aδ-fiber neurons, 178.1 ± 11.08 mV/ms in control, *N *= 18 vs. 171.7 ± 10.97 mV/ms in OA, *N *= 15 (*P *= 0.689). The data are shown in (Fig. 4E).

A similar rationale was adopted to determine the dynamics of repolarization, where AP fall time and maximum falling rate were used to measure the dynamics of the repolarization phase. Repolarization of the AP in either C-fibers or Aδ-fibers in OA animals was not different from that of control animals. AP fall time in C-fiber neurons in control rats (2.6 ± 0.19 ms, *N *= 33) was similar as that in OA rats (3.3 ± 0.62 ms, *N *= 25; *P *= 0.594), as was AP fall time in Aδ-fiber neurons (1.3 ± 0.08 ms, *N *= 18 in control vs. 1.6 ± 0.14 ms, *N *= 15 in OA animals; *P *= 0.084). Maximum falling rate in C-fiber neurons in OA animals (65.1 ± 5.27 mV/ms (*N *= 24) was similar to that in control animals (67.2 ± 4.84 mV/ms; *N *= 33; *P *= 0.734). Maximum falling rate in Aδ-fiber neurons in OA animals (90.5 ± 6.35 mV/ms; *N *= 15) was similar to that in control animals (98.3 ± 5.54 mV/ms; *N *= 18; *P *= 0.364). The data are shown in (Fig. 4F).

Nociceptors typically have a longer afterhyperpolarization period than non-nociceptors [15]. Therefore, measurements of the afterhyperpolarization associated parameters, particularly, 80% afterhyperpolarization recovery time, are liable to be compromised by noise signals during recording. In C-fiber neurons, a total of 16 out of 56 neurons (9 in OA and 7 in control groups) had at least one missing value for afterhyperpolarization, 50% afterhyperpolarization recovery time, or 80% afterhyperpolarization recovery time. A similar problem was observed in Aδ-fiber neuron recordings, where 12 out of 33 neurons (6 in OA and 6 in control groups) lacked the full complement of afterhyperpolarization associated readings. Nonetheless, examination of these afterhyperpolarization associated parameters revealed no difference between OA and control rats, irrespective of the duration or the amplitude. Afterhyperpolarization amplitude was 11.6 ± 0.82 mV (*N *= 30) and 11.9 ± 1.08 mV (*N *= 22) in C-fiber control and OA neurons, respectively (*P *= 0.831), and was 12.1 ± 0.84 mV (*N *= 16) and 10.3 ± 0.99 mV (*N *= 12) in Aδ-fiber control and OA neurons, respectively (*P *= 0.191). The 50% afterhyperpolarization recovery time was 11.4 ± 0.93 ms (*N *= 30 in control) vs. 12.8 ± 1.51 ms (*N *= 21 in OA) in C-fiber neuron group (*P *= 0.414), and was 8.8 ± 1.54 ms (*N *= 16 in control) vs. 10.7 ± 2.28 ms (*N *= 12 in OA) in Aδ-fiber neuron group (*P *= 0.491). The 80% afterhyperpolarization recovery time was 27.5 ± 2.37 ms in control C-fiber neurons (*N *= 24) vs. 30.4 ± 3.16 ms in OA C-fiber neurons (*N *= 18; *P *= 0.451), and was 20.4 ± 2.66 ms in control Aδ-fiber neurons (*N *= 12) vs. (21.1 ± 6.97 ms in OA Aδ-fiber neurons (*N *= 8; *P *= 0.512).

### AP configurations in Aβ-fiber LTMs

For comparison of Aβ-fiber LTMs, 83 such neurons were recorded from 25 naïve control rats and 79 were recorded from 22 OA rats. In terms of the breakdown of different types of Aβ-fiber LTMs, both groups of animals yielded comparable numbers of each neuronal subtype. For example, guard/field hair neurons were recorded from 14 rats in the control group and 15 rats in the OA group. Similarly, muscle spindle neurons (slowly adapting with subcutaneous receptive field) were recorded from 15 control rats and 20 OA rats.

Representative electrophysiological parameters of control A-fiber LTMs, such as resting membrane potential, AP duration at base, AP amplitude, maximum rising rate and maximum falling rate, were comparable to what has been reported in vivo [13,14,37], and also similar to what Ma defined in the low threshold mechanoreceptor category in an in vitro recording that allowed activation of peripheral receptive fields [24]. Electrophysiological parameters of the control neurons were also in the range of what has been reported in medium to large size neurons in vitro [25,26,38,39].

Receptive fields and sites of activation of Aβ-fiber LTMs studied were found throughout the entire hind leg. In the naïve control rats, receptive fields of 55.4% of all of A-fiber LTMs with identifiable receptive fields were on the foot, 19.3% on the calf, 20.5% on the thigh, 1.2% on the ankle joint and 3.6% on the knee joint. In the OA rats, the distribution was as follows: foot (50%), calf (31.8%), thigh (9.1%), ankle joint (3.8%) and knee joint (5.3%). Table 1 summarizes the locations of the receptive fields associated with each neuron type recorded. Some neuronal subtypes only innervated the foot, such as glabrous skin type of rapidly adapting neurons and slowly adapting neurons. Based on our observations, cutaneous rapidly adapting neurons could only be activated by stimulating the glabrous skin of the paw and slowly adapting neurons only by stimulating narrow skin strips surrounding the nails. Receptive fields of the remainder of the neuronal subtypes (i.e. guard/field hair neurons, the Pacinian type of rapidly adapting neurons and muscle spindle neurons) were found ubiquitously innervating the hind leg. The guard/field hair and muscle spindle neuron subgroups contributed the most to the thigh-to-calf receptive fields following joint derangement. In control rats, 9% of guard/field hair neurons and 54% of muscle spindle neurons projected to the calf region, while in OA rats the percentages were 27% in guard/field hair neuron subgroup and 74% in muscle spindle subgroup. Interestingly, the percentages of guard/field hair and muscle spindle neurons projecting to the thigh region was 30% and 36%, respectively, in control rats, but only 7% and 14%, respectively, in OA rats.

**Table 1:** Locations of receptive fields of Aβ-fiber low threshold mechanoreceptors recorded in both the OA rats and the naive control rats

Locations	Foot	Calf	Thigh	Ankle joint	Knee joint
**G/F CTL (*N*)**	14	2	7	1	/
**G/F OA (*N*)**	20	8	2	2	4
**RA CTL (*N*)**	25	2	2		2
**RA OA (*N*)**	34	3	4	3	3
**MS CTL (*N*)**	2	12	8	/	1
**MS OA (*N*)**	5	31	6	/	/
**SA CTL (*N*)**	5	/	/	/	/
**SA OA (*N*)**	7	/	/	/	/
**Aβ LTM CTL (*N*)**	46	16	17	1	3
**Aβ LTM OA (*N*)**	66	42	12	5	7

The locations of receptive fields of neurons included are summarized. The classification adopts only the major anatomical regions, including foot, calf, thigh, ankle joint and knee joint. Abbreviations: CTL, naïve control; OA, osteoarthritis; G/F, neurons which include field neurons and guard hair neurons; RA, rapidly adapting neurons, which include glabrous RA neurons and Pacinian neurons; MS, muscle spindle neurons; SA, slowly adapting neurons; Aβ LTM, Aβ-fiber low threshold mechanoreceptors which include G/F, RA, MS, & SA neurons.

In general, the dynamics of AP genesis were slower in the OA animals, particularly in the depolarization phase of the AP. The duration of the AP was longer in A-fiber LTMs in animals following knee derangement. Compared with the control group (1.0 ± 0.03 ms; *N *= 83), the AP duration at base was significantly wider in the OA group (1.1 ± 0.02 ms; *N *= 79; *P *= 0.028; Fig. 5A). AP half width was significantly longer in neurons in OA animals (0.4 ± 0.02 ms, *N *= 83 in control vs. 0.5 ± 0.01 ms, *N *= 79 in OA; *P *= 0.046). In contrast to the control group (0.4 ± 0.01 ms; *N *= 83), AP rise time was significantly longer in the OA group (0.5 ± 0.01 ms; *N *= 79; *P *< 0.001; Fig. 5B). Maximum rising rate was 291.9 ± 9.41 mV/ms in the control group (*N *= 83), which was significantly faster than in the OA group (254.3 ± 6.22 mV/ms, *N *= 79; *P *= 0.001; Fig. 5E).

However, the AP fall time was not significantly different between the control group and the OA group (*P *= 0.262); readings were 0.6 ± 0.02 ms (*N *= 83) in the control group and 0.6 ± 0.02 ms (*N *= 79) in the OA group (Fig. 5C). Maximum falling rate was similar in the control and the OA group, at 173.3 ± 8.77 mV/ms (*N *= 82) vs. 145.1 ± 4.39 mV/ms (*N *= 79), respectively (*P *= 0.114; Fig. 5F). The AP amplitude was not different in the control vs. the OA group (58.9 ± 1.10 mV, *N *= 83 vs. 57.6 ± 0.98 mV, *N *= 79, respectively; *P *= 0.527; Fig. 5D). The remaining parameters, including conduction velocity, resting membrane potential, 50% afterhyperpolarization recovery time and 80% afterhyperpolarization recovery time, were also not different between the two groups (data not shown).

### Changes in AP configuration in subgroups of Aβ-fiber LTMs

Further comparison was made between the OA group and the control group for each subset of Aβ-fiber LTMs based on the 4 subsets described above: guard/field hair, rapidly adapting, slowly adapting and muscle spindle neurons. Muscle spindle neurons were the most affected, followed by guard/field hair neurons. Surprisingly, no significant difference was identified between control and OA groups in either the rapidly adapting neurons or the slowly adapting neurons. The relatively small number of slowly adapting neurons may have contributed to the lack of a significant difference between the OA neurons and the control neurons.

In muscle spindle neurons, the slower dynamics of the AP was the most obvious of all of the parameters studied. Compared with 0.8 ± 0.06 ms (*N *= 23) in the control group, AP duration at base was significantly wider in the OA group (0.9 ± 0.04 ms, *N *= 24; *P *= 0.04). The AP rise time was 0.3 ± 0.02 ms in control (*N *= 23), which is significantly shorter than that in the OA group (0.4 ± 0.02 ms, *N *= 24; *P *= 0.002). Correspondingly, the maximum rising rate was significantly decreased in OA (307.2 ± 19.73 mV/ms, *N *= 23 in control vs. 246.8 ± 11.34 mV/ms, *N *= 24 in OA; *P *= 0.01). The remaining parameters were not different between the OA and the control groups.

In guard/field hair neurons, the slowing of the AP rise time in OA rats was the only statistically significant change that related to the duration of the AP (0.4 ± 0.02 ms, *N *= 24 in control vs. 0.5 ± 0.02 ms, *N *= 20 in OA; *P *= 0.038). No other significant difference was identified in the remaining parameters related to the duration of AP, such as AP duration at base, AP half width and AP fall time. The resting membrane potential of neurons in the control neurons (-66.3 ± 1.55 mV, *N *= 23) was significantly less depolarized than that of the OA group (-61.1 ± 2.15 mV, *N *= 19; *P *= 0.047).

## Discussion

In the present *in vivo *study using intracellular recording techniques in rat DRG neurons, the electrophysiological properties of Aβ-fiber LTMs (non-nociceptive) and C- and Aδ-fiber nociceptive primary sensory neurons were systematically evaluated in a rat model of OA at one month following model induction. This model was confirmed with fully established osteoarthritis characteristics, namely characteristic cartilage degeneration within the knee joint, edema, increased permeability of the knee vasculature and tactile hypersensitivity of the affected lower limb.

Several important observations were made in the electrophysiological studies. There were prominent changes in electrophysiological properties of Aβ-fiber LTMs suggesting a slowing of the dynamics of AP generation, including a wider duration of the AP and a slower maximum rising rate. Importantly, even Aβ-fiber LTMs innervating non-articular structures were affected by the injury initiated in the knee joint. It is not clear what is driving the changes in non-nociceptive neurons or how these neurons are preferentially affected, but the coincidence of changes in nociception and the selective changes in these neurons might imply a role of Aβ-fiber LTMs in the pathogenesis of OA pain.

In contrast, no changes were observed in Aδ- or C-fiber neurons, except a more depolarized Vm in Aδ neurons. This lack of change in the functional properties was surprising, given the changes in these neurons in animal models of inflammatory pain, as discussed below. However, caution should be reminded to consider the alone-standing depolarized Vm in Aδ neurons as a proof of lowered activation threshold in these neurons and consequently as a mechanism of joint pain, as additional evidence of altered AP genesis in these neurons could not be found.

### Joint vs. non-joint afferents

Accumulating clinical data and our own observations suggest sensory neuron changes beyond simply changes in knee joint nociceptors, and these may relate to OA pain pathogenesis. This study was initially designed to investigate changes in DRG neurons that can be activated by stimulating knee joint structures, such as knee joint ligaments, muscle attached to the joint and skin covering the joint. Unexpectedly, during pilot studies other sensory neurons within the same DRG, with receptive fields far beyond the knee joint such as foot, seemed to have undergone changes. This observation is consistent with pain referred to other areas beyond the joint, as reported in OA patients [6,40]. Thus, we felt compelled to change our initial experimental design and to include in this study all sensory neurons regardless of their receptive fields within L_4 _DRG, which is known as having the largest number of knee joint afferents [41]. One might argue in favor of recording from articular nerve to achieve the highest yield of knee joint nociceptors in order to comment on the role of these neurons. However, mounting evidence has shown that C- or Aδ-fiber joint nociceptors are not necessarily the cause of OA pain. For example, there is limited correlation of the severity of joint pathology with the severity of joint pain [42-44], suggesting that non-articular factors may give rise to OA pain pathogenesis. Further, in approximately 12% of patients, joint pain is not relieved by total joint replacement [7], and therefore this post-replacement pain cannot be maintained by sensitization or activation of nociceptors in the joint. Our observations are in line with these clinical presentations in OA patients. We report here that the most significant change in properties was in non-knee joint afferents and between Aβ-fiber low threshold non-nociceptive neurons and small diameter C- or Aδ-fiber "pain" neurons.

### The neuropathic pattern of affected neuronal types in OA

The pattern of prominent changes in large Aβ-fiber neurons and the lack of change in small C-fiber neurons is commonly reported in neuropathic models of chronic pain [20-26], and is rarely seen in inflammation models of chronic pain [13,19]. Therefore, we propose that the electrophysiological changes in our OA rats may be associated with a neuropathic etiology that follows model induction. OA rats exhibited signs of movement-evoked pain behavior as suggested by the tail flick results in these animals. Pain behaviors in our OA animals and characteristic movement-evoked pain in OA patients resemble the movement-evoked pain that has been reported in bone cancer pain animals, a complex pain condition that is believed to involve neuropathic mechanisms and large diameter Aβ-fiber neurons [45,46]. Further, our results and interpretation are consistent with the suggestion of Ivanavicius et al. (2007), who referred to OA pain as having a neuropathic component in view of the mild immune cell infiltration and the poor efficacy of non-steroidal anti-inflammatory drugs [47], and are consistent with the clinical picture of OA patients who commonly describe their pain with terms typically associated with neuropathic pain processes [40].

This prompts a comparison of our data with data from other groups investigating changes in DRG neurons in models of peripheral neuropathic pain vs. models of peripheral inflammatory pain. It has been suggested that inflammation and neuropathic etiologies likely affect distinct populations of DRG neurons in various chronic pain models. In superficial inflammation models, for example as induced by injecting complete Freund's adjuvant subcutaneously [13,19], only Aδ-fiber neurons and C-fiber neurons undergo significant changes in electrophysiological properties, with changes in C-fiber neurons being more severe. In adjuvant-induced joint inflammation, to our knowledge no studies on the properties of DRG neurons are available. However, indirect evidence has suggested that the effects of joint inflammation do not influence the large, non-nociceptive A-fiber neurons, as indicated by the lack of expression of two pain-related peptides, calcitonin gene-related peptide and substance P in those neuronal types [48,49]. On the contrary, in classic neuropathic models, such as the complete sciatic nerve transection model [20], the partial sciatic nerve transection model [22], and the lumbar spinal nerve transection model [21,23-26], changes in AP configuration in A-fiber neurons characterize changes in primary sensory system, and are common. Although in some studies on neuropathic models [20,21,24] changes in C-fiber neurons have been reported, such changes are less prominent than those in A-fiber neurons.

### Functional changes in Aβ-fiber LTMs

What prompts us to question the participation of C- or Aδ-fiber neurons in OA pain is the lack of correlation of the various changes in nociception with changes in the function of these neurons. It is obvious that some other mechanisms should account for the changes in nociception, including lowered activation threshold of the hind paw and painful flexion and extension of the affected knee joint. These changes in nociception, mainly mechanical sensitivity, occur along with changes in functional properties of Aβ-fiber LTMs. Studies from other research groups also suggest a possible role of Aβ-fiber LTMs in sensory deficits, such as allodynia [20,21,23-25,50], although detailed mechanisms have not been identified. One possible explanation is that some Aβ-fiber non-nociceptive neurons take up a new role in nociception and begin to convey signals along novel pathways leading to nociception/pain after model induction [51].

The observed changes in AP configuration in Aβ-fiber non-nociceptor neurons, including wider AP duration, longer AP rise time and slower maximum rising rate, reflect slowed dynamics of depolarization and therefore suggest a change in sodium currents in these neurons, either a functional change or a change in expression. However, the specific ionic mechanisms remain unknown, partly because details of the specific sodium channel composition has not yet been identified in functionally classified sensory neuron subtypes, such as hair, Pacinian, glabrous rapidly adapting or muscle spindle neurons (all examples of Aβ-fiber LTMs). According to a recent paper by Fukuoka et al., large A-fiber neurons are thought to express both TTX-sensitive sodium channels (Nav 1.1, Nav 1.6 and Nav 1.7) and TTX-resistant sodium channels (Nav 1.8, Nav 1.9) [52]. Moreover, after axotomy, 75% of A-fiber neurons re-express the embryonic TTX-sensitive Nav 1.3 channel [52]. Therefore, the significance of our data should also be considered in the context of the possible summation effect of various changes in sodium channels Nav 1.1, Nav 1.3, Nav 1.6, Nav 1.7, Nav 1.8, and Nav 1.9.

## Conclusion

The patterns of the changes in the electrophysiological properties of Aβ-fiber LTMs but not in C- or Aδ-fiber neurons are consistent with observations from other laboratories in models of peripheral neuropathy but not models of peripheral inflammation. These changes might reflect a change in functional role of primary afferent sensory processing, which might then constitute a novel mechanism in the pathogenesis of pain at the early phase of OA.

## Competing interests

The authors declare that they have no competing interests.

## Authors' contributions

JLH conceived of, designed, and coordinated the study. QW did the electrophysiological experiments, analyzed the data and performed statistical analyses. QW wrote the initial draft of the manuscript. Both authors worked on refining this draft and the revision based on editorial review. Both authors have read and approved the final manuscript.
